# Latent class analysis for health-related quality of life in nurses in China

**DOI:** 10.3389/fpubh.2024.1433018

**Published:** 2024-12-11

**Authors:** Yan Zhao, Bei Yang, Jianying Chu

**Affiliations:** ^1^Department of General Surgery, Qilu Hospital of Shandong University, Jinan, China; ^2^Department of Plastic, Aesthetic, Reparative and Reconstructive Unit Nursing, West China Second Hospital, Sichuan University/West China School of Nursing, Sichuan University, Key Laboratory of Birth Defects and Related Diseases of Women and Children (Sichuan University), Ministry of Education, Chengdu, Sichuan, China; ^3^Department of Obstetrics, Qilu Hospital of Shandong University, Jinan, Shandong, China; ^4^Nursing Theory & Practice Innovation Research Center, Shandong University, Jinan, Shandong, China

**Keywords:** nursing staff, quality of life, EQ-5D, latent class analysis, psychological distress

## Abstract

**Background:**

This study aimed to identify the types of quality of life (QoL) based on the five dimensions of the EQ-5D and predict factors affecting QoL.

**Methods:**

A multistage stratified cluster sampling survey was conducted among the staff of 12 general hospitals, 1,965 nurses completed the survey, and the data were analyzed using SPSS 26.0 and Mplus 8.3 for latent analysis.

**Results:**

Three latent classes of QoL were identified: low-level (2.8%), pain and discomfort (7.6%), medium-level (47.1%), and high-level (42.5%). The types and characteristics of QoL differed among these latent classes. The low-level group had the lowest EQ visual analog scale (EQ-VAS) score (*F* = 75.217, *P* < 0.001) and the highest K10 score (*F* = 61.90, *P* < 0.001). Moreover, increased age (*OR* = 0.819, 95% *CI*: 0.817–0.973), never having drunk alcohol (OR = 0.107, 95% CI: 0.023, 0.488), and increased EQ-VAS scores (*OR* = 0.935, 95% *CI*: 0.919, 0.952) were protective factors for quality of life, while working in obstetrics and gynecology (*OR* = 6.457, 95% *CI*:1.852, 22.512) and higher K10 scores (*OR* = 1.153, 95% *CI*: 1.100, 1.209) were risk factors for quality of life.

**Conclusion:**

The results indicated significant heterogeneity in the types of QoL and identified predictors of QoL. These findings provide basic information for the development of nursing interventions to improve quality of life and identified specific characteristics that should be considered during intervention development.

## 1 Background

Health-related quality of life refers to the experience of individuals in different cultures and value systems regarding their goals, expectations, standards, and living conditions related to things they care about as a subjective perception and expression of health; it reflects individuals' physical, psychological, social, spiritual, and personal roles (1). The complete state of experience in aspects such as psychology, society, spirit, and personal role function is a comprehensive reflection of an individual's health status (2).

There are several factors influencing health-related quality of life, which are not only related to individual health status, health behavior, economic, and cultural factors (2–5), but also closely related to occupational characteristics (6). Nursing staff play an important functional role in medical and health services, and the health-related quality of life of clinical nurses is affected by the high risk and pressure of nursing work and the insufficiency of family and social resources (7). Studies have shown that nurses' quality of life is at the middle to lower levels (6). Nurses' quality of life is important as it is closely related to the quality of nursing work (8).

The Latent Class Model (LCM) is a statistical analysis method in which the model fit is determined by the data, which can capture the inequalities of the population, thereby objectively identifying mutually exclusive subtype categories (9). From the group heterogeneity perspective, this study explored the latent categories of nurses' quality of life based on the LCM and identified the performance characteristics of different latent categories regarding demographic sociology, physical health, mental health, etc. Through population characteristics, low-level QoL groups can quickly be identified, effective targeted intervention strategies can be formulated, and a theoretical basis for improving nurses' QoL can be provided.

## 2 Methods

### 2.1 Study population

The study sample consisted of 1,965 nurses employed at general hospitals in Shandong Province. A multi-stage stratified whole-group sampling method was applied from November 2020 to January 2021. First, all prefecture-level cities in Shandong Province were ranked according to their 2018 GDP per capita levels and were divided into three categories: good, medium, and poor. Subsequently, one prefecture-level city was selected from each category via simple random sampling (Qingdao City, Zaozhuang City, and Dezhou City). Second, a district city with only one municipal general hospital was surveyed. In cases of more than one hospital being present within a district city, one general hospital was selected via simple random sampling. Following this, three counties (districts/county-level cities) in each prefecture-level city were randomly selected. In each county (district/county-level city), one (district/county-level city) general hospital was randomly selected via the same sampling method used for city-owned hospitals. As such, this study surveyed 12 general hospitals, including three city-affiliated and nine county-affiliated hospitals. Among these, three wards were randomly selected for each discipline according to the discipline classification (all wards were surveyed in cases where fewer than three wards existed). Further, the surveyed wards' night nursing staff members were asked to complete the questionnaire.

This study was reviewed and approved by the Ethics Committee of the School of Public Health, Shandong University (approval number: 20181219). The fieldwork was initiated after approval was obtained. All participants signed an informed consent form before participating in the survey.

### 2.2 Quality of life

The EQ-5D-3L was used to assess QoL in this study. The scale includes two parts: the EQ-5D descriptive system and EQ-VAS (10). The health description system includes five dimensions: mobility, self-care, daily activities, pain/discomfort, and anxiety/depression, with three levels in each dimension: difficulty/problem, moderate difficulty/problem, and severe difficulty/problem; the EQ-VAS section is a vertical visual scale with 100 at the top representing “best health” and 0 at the bottom representing “worst health.” This study calculated the utility value of the EQ-5D using the Chinese version of the TTO (11).

### 2.3 Psychological distress

Kessler Psychological Distress Scale (K10) is a 10-item screening tool designed for non-specific psychological distress (12). In our survey, it was measured by the Chinese version of the K10 (13). Each item adopts a 5-level score of 1 (almost never) to 5 (all the time), with a total score of 50. A score of >15 indicates psychological distress, 10–15 indicates mild distress, 16–21 indicates moderate distress, 22–29 indicates severe distress, and 30–50 indicates severe psychological distress. The Cronbach's alpha coefficient in this study was 0.930.

### 2.4 Statistical analyses

SPSS 25.0 and Mplus 8.3 statistical software were used for data entry and statistical analysis. Enumeration data were expressed as frequency (percentage), measurement data were normally distributed, and expressed as mean ± standard deviation. Latent class analysis was used to classify participants' quality of life responses. When performing latent class analysis, the evaluation indicators of model adaptation include (1) Akaike Information Criteria (AIC), Bayesian Information Criteria (BIC), and sample size-adjusted BIC (saBIC). The smaller the value, the better the fit and the higher the performance of the model. (2) Entropy index: the higher the entropy value, the higher the accuracy of classification; when entropy = 0.6, the classification accuracy exceeds 80%, and when entropy = 0.8, the classification accuracy exceeds 90%. (3) The likelihood ratio test (Lo-Mendell-Rubin, LMR) and Bootstrap-based likelihood ratio test (BLRT). When the *P* value reaches a level of significance, it indicates that the model of k categories is significant due to k-1 class models (14, 15). When the models of the evaluation indicators were inconsistent, this study comprehensively considered the results of measuring each indicator and combined the “best explanation model” to determine the best model. Multivariate logistic regression was used to analyse the factors influencing each category. All statistical tests were two-sided with a test level of α = 0.05.

## 3 Results

### 3.1 The current status of the quality of life of nurses

According to the EQ-5D Effect Value Score System calculation in China, the EQ-5D and its health effect value of the nurse group was (0.9382 ± 0.1078), which was significantly lower than the QoL level of urban civilian in a certain province of China (16) (*t* = −7.125, *P* < 0.001).

### 3.2 Classification of nurses' quality of Life

This study begins with the initial model of the two categories and gradually increases the number of categories to determine the best model. The fitting index data are presented in Table 1. As the model category increased, the AIC, BIC, and SaBIC values of each category decreased. When the number of categories was three, the entropy value of the latent-category model was 0.804. When the number of categories was four, the entropy value was 0.784, nearly 0.8, which indicates that the classification accuracy exceeds 90%. When dividing the categories, they were distinguished in combination with practical significance and interpretability. The second category was too concise and lacked practical significance, while the fifth category was too complicated to explain. The AIC and BIC indicators of the fourth category were smaller than those of the third category, and the entropy value was close to 0.8. Comprehensively, the four-category model was the optimal model.

**Table 1:** Fit indices of latent class analysis and distribution rate of HRQoLs (EQ-5D).

**Cluster**	**AIC**	**BIC**	**SaBIC**	**Entropy**	**LMR**	**BLRT**	**Latent class distribution rate (%)**				
							**1**	**2**	**3**	**4**	**5**
2-class	30,721.99	30,852.45	30,772.30	0.819	< 0.001	< 0.001	11.9	88.1			
3-class	30,562.57	30,758.25	30,643.88	0.804	0.017	< 0.001	48.9	4.2	46.9		
4-class	30,467.94	30,728.84	30,576.35	0.784	0.042	< 0.001	2.8	7.6	47.1	42.5	
5-class	30,406.42	30,732.55	30,541.94	0.783	0.002	< 0.001	11.6	37.9	1.5	44.0	5.0

**Figure 1:**
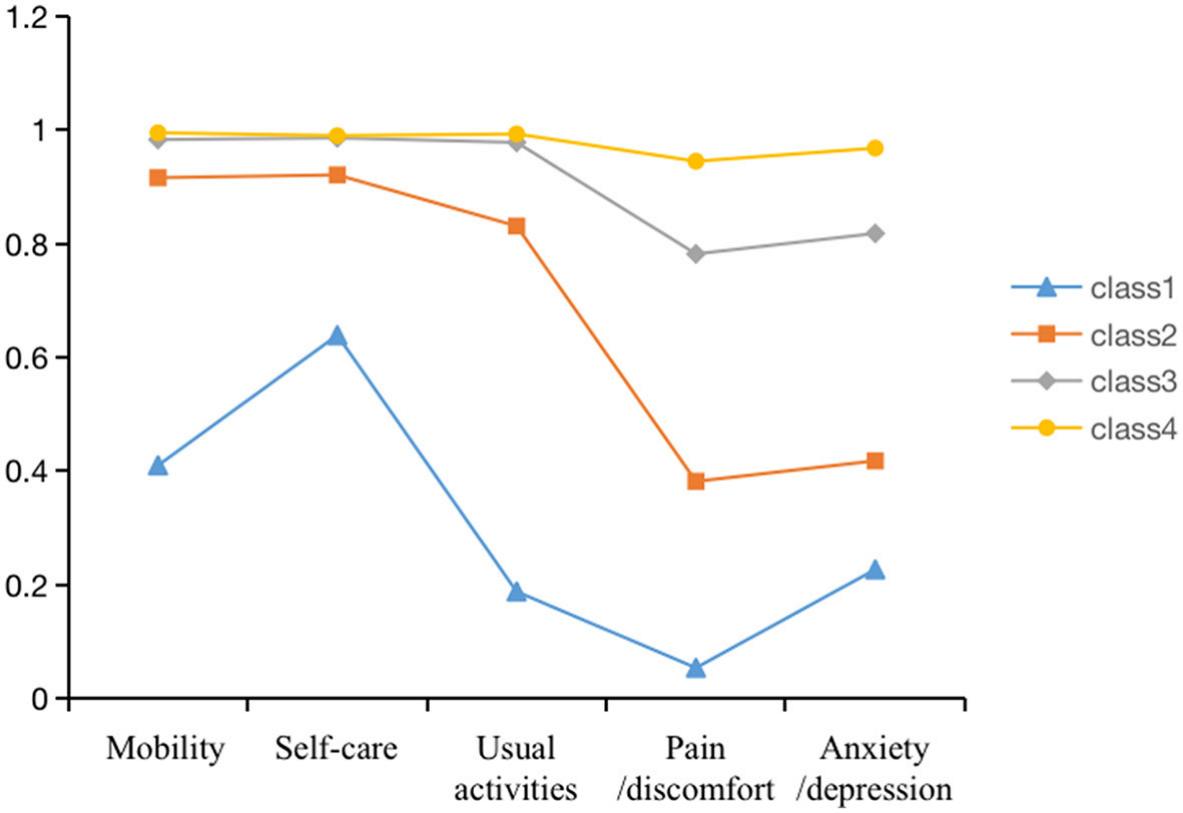
Types of health-related quality of life by latent classes.

A model with four potential categories was used as the optimal model to obtain the parameter estimation results. The conditional probability distributions for each category are presented in Figure 1. In this study, nurses were divided into four groups based on five factors. In category 1, the conditional probabilities of mobility, self-care, daily activities, pain, and anxiety/depression at the no difficulty level were 0.410, 0.639, 0.188, 0.054, and 0.227, respectively; in category 2, the conditional probabilities were 0.916, 0.921, 0.831, 0.382, and 0.418, respectively; in category 3, the conditional probabilities were 0.983, 0.986, 0.978, 0.782, and 0.818, respectively; in category 4, the conditional probabilities were 0.995, 0.990, 0.993, 0.945, and 0.968, respectively. The distribution trend of each factor was relatively consistent, consequently, they were named: “Low-level group (2.8%) (category 1),” “Pain and discomfort group (7.6%) (category 2),” “Medium-level group (47.1%) (category 3),” and “High-level group (42.5%) (category 4).”

### 3.3 Comparison of various demographic and psychological characteristics among various categories

Comparing different category characteristics, the results showed that the average age of the low-level group was (31.42 ± 5.68) years old, which was lower than other groups, and the proportion of junior professional titles and below was 79.2%, which was higher than other groups.

The proportion of obstetrics and gynecology workers was 31.3%, which was higher than other groups. In addition, a one-way analysis of variance was used to explore the differences in EQ-VAS scores and K10 scores for each category. The results showed that the low-level group had the lowest EQ-VAS score, that is, the worst health level (*F* = 75.217, *P* < 0.001), and the highest K10 score, that is, the worst psychological health level (*F* = 61.90, *P* < 0.001) (Table 2).

**Table 2:** Comparison of characteristics among latent classes in Chinese nurses *n* (%).

**Variables**	**Class 1 (*n =* 48)**	**Class 2 (*n =* 129)**	**Class 3 (*n =* 799)**	**Class 4 (*n =* 719)**	**F/χ^2^**	***P***
**Personal characteristics**						
Age (years, M ± SD)	31.42 ± 5.68	35.87 ± 7.17	30.50 ± 6.38	36.16 ± 7.56	91.067	< 0.001
**Gender**						
Male	2 (4.2)	5 (3.9)	50 (6.3)	16 (2.2)	15.404	0.001
Female	46 (95.8)	124 (96.1)	749 (93.7)	703 (97.8)		
**Marital status**						
Unmarried	8 (16.7)	15 (11.6)	224 (28.0)	45 (6.3)	140.957	< 0.001
Married	538 (79.2)	114 (88.4)	568 (71.1)	664 (92.4)		
Other	2 (4.2)	0 (0)	7 (0.9%)	10 (1.4%)		
**Education level**						
Master and above	1 (2.1)	4 (3.1)	14 (1.8)	15 (2.1)	32.873	< 0.001
Undergraduate	43 (89.6)	107 (82.9)	595 (74.5)	607 (84.4)		
Below undergraduate	4 (8.3)	18 (14.0)	190 (23.8)	97 (13.5)		
**Professional title**						
Associate senior and above	1 (2.1)	14 (10.9)	7 (0.9)	62 (8.6)	214.873	< 0.001
Intermediate	9 (18.8)	59 (45.7)	137 (17.1)	297 (41.3)		
Primary and below	38 (79.2)	56 (43.4)	655 (82.0)	360 (50.1)		
**Department**						
Internal medicine	9 (18.8)	26 (20.2)	202 (25.3)	216 (30.0)	23.438	0.024
Surgery	13 (27.1)	33 (25.6)	178 (22.3)	157 (21.8)		
Obstetrics and Gynecology	15 (31.3)	27 (20.9)	132 (16.5)	111 (15.4)		
Pediatrics	7 (14.6)	16 (12.4)	101 (12.6)	98 (13.6)		
Other	4 (8.3)	27 (20.9)	186 (23.3)	137 (19.1)		
**Health status**						
EQ-VAS (M ± SD)	54.58 ± 17.22	67.26 ± 17.48	78.73 ± 17.20	83.40 ± 15.05	75.217	< 0.001
**Health behavior**						
**Smoking**						
No	46 (95.8)	122 (94.6)	785 (98.2)	707 (98.3)	9.200	0.027
Yes	2 (4.2)	7 (5.4)	14 (1.8)	12 (1.7)		
**Drinking**						
No	43 (89.6)	124 (96.1)	768 (96.7)	705 (98.5)	15.714	0.001
Yes	5 (10.4)	5 (3.9)	26 (33)	11 (1.5)		
**Psycho-emotional status**						
K10 score (M ± SD)	29.96 ± 7.72	26.65 ± 7.22	22.80 ± 7.37	20.05 ± 6.39	61.90	< 0.001

### 3.4 Multivariate logistic regression analysis of potential quality of life categories of nurses

Nurses' quality of life category was taken as the dependent variable (category four as the reference group), nurses' characteristics (age, sex, marital status, education level, professional title, and department), health level (EQ-VAS), health behavior (smoking, alcohol consumption), and psycho-emotional status (K10 score) were taken as independent variables, and a multivariate logistic regression model was constructed to analyse the relevant factors influencing nurses' low health levels. The results showed that increased age (*OR* = 0.819, 95% *CI*: 0.817, 0.973), never having drunk alcohol (*OR* = 0.107, 95% *CI*: 0.023, 0.488), and increased EQ-VAS scores (*OR* = 0.935, 95% *CI*: 0.919, 0.952) were quality of life protective factors, while working in obstetrics and gynecology (*OR* = 6.457, 95% *CI*: 1.852, 22.512) and higher K10 scores (*OR* = 1.153, 95% *CI*: 1.100, 1.209) were quality of life risk factors (Table 3).

**Table 3:** Multinomial logistic regression models of latent class analysis of health-related quality of life.

**Variables**	**Class 1**		**Class 2**		**Class 3**	
	**OR**	**95% CI**	**OR**	**95% CI**	**OR**	**95% CI**
**Personal characteristics**						
Age	**0.891**	**0.817–0.973**	**0.951**	**0.913–0.991**	**0.927**	**0.903–0.951**
Gender (Male)	0.982	0.149–6.482	0.972	0.269–3.513	1.474	0.726–2.994
**Marital status**						
Unmarried	0.486	0.053–4.475	1.279	0.742–5.617	**3.374**	**1.034–11.010**
Married	0.371	0.050–2.754	1.733	0.733–8.873	1.252	0.407–3.875
**Education level**						
Master and above	1.846	0.144–23.681	0.839	0.219–3.224	0.897	0.362–2.225
Undergraduate	2.280	0.689–7.544	0.774	0.428–1.401	0.735	0.540–1.001
**Professional title**						
Associate senior and above	1.016	0.093–11.104	**4.619**	**1.687–12.645**	**0.236**	**0.090–0.619**
Intermediate	0.431	0.145–1.282	**2.134**	**1.203–3.787**	**0.629**	**0.450–0.881**
**Department**						
Internal medicine	1.574	0.425–5.825	0.624	0.330–1.180	**0.649**	**0.461–0.914**
Surgery	3.112	0.903–10.725	0.929	0.505–1.710	0.845	0.593–1.203
Obstetrics and Gynecology	**6.457**	**1.852–22.512**	1.311	0.690–2.494	0.879	0.599–1.288
Pediatrics	2.544	0.648–9.992	0.691	0.336–1.423	**0.641**	**0.426–0.964**
**Health status**						
EQ-VAS (M ± SD)	**0.935**	**0.919–0.952**	**0.960**	**0.948–0.971**	**0.989**	**0.982–0.997**
**Health Behavior**						
Smoking (No)	1.324	0.157–11.177	0.295	0.076–1.147	2.265	0.949–7.261
Drinking (No)	**0.107**	**0.023–0.488**	1.124	0.282–4.476	0.451	0.181–1.124
**Psycho-emotional status**						
K10 score (M ± SD)	**1.153**	**1.100–1.209**	**1.096**	**1.065–1.129**	**1.062**	**1.043–1.082**

Criterion variables: economic activities (no = 0), smoking (no = 0), drinking (no = 0), depression (no = 0), suicidal compulsion (no = 0), and chronic disease (no = 0). Bold values represent the statistical significance at the *P*-value < 0.05.

## 4 Discussion

### 4.1 Low level of quality of life for nurses

This study found that the quality of life of nurses was lower than that of the general urban population in China (16). Nurses in the 21^st^ century are facing increasingly difficult challenges to maintain a high quality of life (17). From long hours to inadequate pay, nurses are often overworked and underpaid (18, 19). According to the China Statistical Yearbook on Health and Family Planning, the number of registered nurses in China in 2015 was 3 million (20). Considering China's great population, China needs at least 6 million nurses (21). Due to the shortage of medical personnel, they have to work longer hours under tremendous pressure but receive very low rewards (22, 23). This leads to decreased job satisfaction and a decrease in quality of care. Therefore, there is a need to focus on the professional development of nurses at the policy level, improve the protection and training system and raise the quality of life level of the nursing community.

### 4.2 Potential categories of quality of life levels in the nurse population

This study used latent class analysis to examine the quality of life of the nurse population to reveal differences in the different quality of life categories and their distribution on demographic and sociological variables. The model fitting results of the four latent categories were found to be the best, consequently, the researchers divided quality of life into four categories according to the nurses' response probability map of each item of the EQ-5D-3L scale. These categories were named low-level groups, pain and discomfort group, medium-level group, and high-level group; each group accounted for 2.8%, 7.6%, 47.1%, and 42.5%, respectively, among which the middle- and high-level groups were the majority groups. In addition, the pain symptoms of nurses in each group were more apparent than other symptoms, which may be related to the high work intensity and heavy workload of nurses (24, 25). The results suggest that nursing managers should pay attention to the quality of life and work intensity of nurses and take appropriate timely intervention measures to improve the pain symptoms of clinical nurses.

### 4.3 Attention should be paid to low quality-of-life groups

Compared to the nurses in the high-level group, younger age, working in obstetrics and gynecology, and poor emotional state were quality-of-life risk factors for the nurses in the low-level group, while higher health scores and non-smoking were quality-of-life protective factors for the nurses in the low-level group.

Study have shown that an increase in years of working experience results in employees having strong professional technical abilities, rich knowledge, strong economic strength, and social status (26). Consequently, the level of supervisor happiness is high and the quality of life is better (27), which is consistent with this study's results. Compared to the high-level group, obstetrics and gynecology nurses were 6.45 times more likely to experience a lower quality of life. A possible reason is that the work of obstetrics and gynecology nurses is centered on the puerpera, and the time of puerpera's labor has no rules (28). Consequently, nurses often need to participate in emergency treatment and surgery (29). Therefore, the requirements of obstetrics and gynecology nurses are relatively high. In addition, it is common for obstetrics and gynecology nurses to work overtime. The significant amount of work consumes the obstetrics and gynecology nurses' energy, resulting in poor quality of life (30). Simultaneously, the quality of life of nurses with poor emotional states was lower, while the quality of life of nurses who did not drink alcohol and had a high level of health was higher, further verifying previous research results (27, 31). Therefore, the results suggest that clinical nursing managers should pay attention to low-level quality-of-life nurse groups, such as obstetrics and gynecology nurses with fewer working years.

### 4.4 Limitations

This study has some limitations. Firstly, although this study adopted a multistage stratified whole-group sampling method, the selection of primary hospitals was relatively large, and the representativeness was biased. Therefore, these conclusions should be further explored and tested continuously. Secondly, this study did not include interventional research or an assessment of the impact of psychotherapy or psychological interventions on nurses' quality of life. Considering the potential significance of these interventions, future research is needed to address this gap.

## 5 Conclusions

In this study, the quality of life levels of nurses in 12 hospitals in Shandong province was analyzed using latent class analysis, and the number of optimal model categories was fitted to four groups: low-level group, pain and discomfort group, medium-level group, and high-level group. The clinical characteristics of nurses in different groups were distinguished, and the probability of entering the low-level quality of life group was higher for younger nurses, who work in obstetrics and gynecology, and have poor emotional states than those entering the high-level group. This method sought to identify nursing staff with poorer quality of life in a more targeted manner and can provide an important reference basis for clinical interventions to improve nurses' quality of life.

## Data Availability

The raw data supporting the conclusions of this article will be made available by the authors, without undue reservation.
